# Monoamine Oxidases as Potential Contributors to Oxidative Stress in Diabetes: Time for a Study in Patients Undergoing Heart Surgery

**DOI:** 10.1155/2015/515437

**Published:** 2015-05-25

**Authors:** Oana M. Duicu, Rodica Lighezan, Adrian Sturza, Raluca A. Ceausu, Claudia Borza, Adrian Vaduva, Lavinia Noveanu, Marian Gaspar, Adina Ionac, Horea Feier, Danina M. Muntean, Cristian Mornos

**Affiliations:** ^1^Department of Functional Sciences-Pathophysiology, “Victor Babes” University of Medicine and Pharmacy, 2 Eftimie Murgu Square, 300041 Timisoara, Romania; ^2^Department of Microscopical Morphology-Histology, “Victor Babes” University of Medicine and Pharmacy, 2 Eftimie Murgu Square, 300041 Timisoara, Romania; ^3^Department of Microscopical Morphology-Morphopathology, “Victor Babes” University of Medicine and Pharmacy, 2 Eftimie Murgu Square, 300041 Timisoara, Romania; ^4^Department of Cardiology-Cardiovascular Surgery, “Victor Babes” University of Medicine and Pharmacy, 2 Eftimie Murgu Square, 300041 Timisoara, Romania; ^5^Department of Cardiology-2nd Cardiology Clinic, “Victor Babes” University of Medicine and Pharmacy, 2 Eftimie Murgu Square, 300041 Timisoara, Romania

## Abstract

Oxidative stress is a pathomechanism causally linked to the progression of chronic cardiovascular diseases and diabetes. Mitochondria have emerged as the most relevant source of reactive oxygen species, the major culprit being classically considered the respiratory chain at the inner mitochondrial membrane. In the past decade, several experimental studies unequivocally demonstrated the contribution of monoamine oxidases (MAOs) at the outer mitochondrial membrane to the maladaptative ventricular hypertrophy and endothelial dysfunction. This paper addresses the contribution of mitochondrial dysfunction to the pathogenesis of heart failure and diabetes together with the mounting evidence for an emerging role of MAO inhibition as putative cardioprotective strategy in both conditions.

## 1. Introduction

According to the World Health Organization, cardiovascular diseases represent the number one cause of death globally (WHO March 2013). In particular, coronary heart disease is a leading cause of mortality and morbidity due to heart failure (HF). With an increasingly aging population and improved survival after the onset of HF in elderly, the syndrome is recognized as a growing problem for the health-care systems worldwide due to its enormous financial burden [1]. Diabetes mellitus (DM), the most severe metabolic disease, is currently viewed as a serious threat to global health due to its increasing prevalence, especially in developing countries; it is predicted that 592 million people will have diabetes by 2035 [2]. The association of type 2 DM with increased cardiovascular morbidity and mortality is widely recognized [3] with both traditional and nontraditional risk factors being involved [4]. This is particularly true for the association between HF and diabetes, since according to the Framingham Study the frequency of HF was significantly higher in diabetic patients (mainly in women) as compared to the age-matched healthy subjects [5]. In the past two decades, mounting epidemiological and clinical evidence suggests that DM increases the risk for the so-called “diabetic cardiomyopathy” that develops independently of other risk factors such as coronary disease and hypertension [6].

Oxidative stress is the common pathomechanism that greatly influences the progression of both cardiovascular and metabolic diseases. The difficulty to assess the redox pathophysiology is related to both its spatiotemporal heterogeneity and the existence of complex networks of redox signaling as well as the amplification of ROS generation that occur in pathological conditions. This latter condition, known as either “ROS-induced ROS release” [7, 8] or the “kindling radicals” concept [9, 10], refers to the situation in which extramitochondrial (or even mitochondrial) ROS are acting mainly as triggers for mitochondrial ROS production. From mechanistic point of view, this crosstalk to and from mitochondria [9] renders the complete characterization of a pathological entity in a particular model when using the causal reasoning difficult [11]. However, from therapeutic point of view, this crosstalk among several ROS generators appears to be advantageous since there is sound experimental evidence for the partial or even complete abrogation of oxidative stress (and of its deleterious consequences) by inhibiting a single source of ROS [12]. The complexity of the prooxidative status in patients with HF and DM is further contributed by the chronic low-grade inflammation with the induction of a vicious, self-perpetuating circle, responsible for the: (i) aggravation of the oxidative stress via ROS generation by the activated monocytes/macrophages [13–15] that can also interact with cardiac cells [16] and (ii) activation of the inflammasome and phagocytes by ROS originating in cardiac mitochondria [17–20].

The prominent sources of cardiovascular oxidative stress in HF and DM are mitochondria, uncoupled eNOS, and nicotinamide adenine dinucleotide phosphate (NADPH, Nox) oxidases (reviewed in [11, 21–25]). Whereas in case of Nox conflicting data have been reported in the literature, with both protective [26] and deleterious [27] roles of Nox4-derived ROS in the development of HF, the contribution of mitochondria and eNOS as major sources of intracellular oxidative stress is a widely accepted concept [10, 28–30]. However, in the past decade, the contribution of monoamine oxidases (MAOs), FAD-containing dehydrogenases located at the outer mitochondrial membrane, as novel sources of obligatory ROS generation in the cardiovascular pathology, has become evident [31].

Here, we briefly review the contribution of mitochondrial dysfunction to the pathogenesis of heart failure and diabetes, pointing out the commonalities between these two conditions. We will further refer to the beneficial effects of MAO inhibition in relation to cardiovascular pathology and experimental diabetes. Finally, we will emphasize the need for a translational approach, assessing the contribution of MAO-related oxidative stress to the pathogenesis of mitochondrial, endothelial, and contractile dysfunction in diabetic* versus* nondiabetic patients undergoing heart surgery.

## 2. Mitochondrial Dysfunction in Heart Failure

Mitochondria, the powerhouses of our cells that provide the main amount of energy required for normal cardiomyocyte function, have emerged in the past decades as the major sources and amplifiers of oxidative damage in the cardiovascular system [32].

Heart failure is a multietiological clinical syndrome that develops progressively as a consequence of a primary cause (acute or chronic) that impairs the systolic function (HF with reduced ejection fraction) and/or the diastolic one (HF with preserved ejection fraction). In the vast majority of cases, the primary event is represented by either a chronic hemodynamic (pressure or volume) overload or an acute coronary syndrome that triggers the pathological hypertrophy and ultimately the development of HF. Several experimental models (mainly mechanically or genetically induced hypertension and coronary artery ligation) have been used to mimic these conditions and shed light on the pathophysiology of the syndrome.

In the past decade, mitochondrial dysfunction and the subsequent disrupted redox signaling have been systematically reported to underlie both the development of pathological ventricular hypertrophy and its progression towards the overt cardiac failure (reviewed in [21, 22, 33, 34]). At variance from physiological (adaptive) hypertrophy, where mitochondrial function increases in order to maintain adequate cardiac function [35], pathological (maladaptive) hypertrophy and heart failure have been reported to share similar mitochondrial abnormalities [22] with respect to (i) substrate metabolism (decreased fatty acid oxidation plus increased/unchanged or decreased glucose oxidation in advanced stages of HF, responsible for the energetic deficiency [25]), (ii) calcium handling [36, 37], (iii) respiratory function (decreased in most of the cases; see below), and (iv) ROS production (variable degrees of oxidative stress [38]).

The contribution of metabolic impairment with the subsequent energetic dysfunction to the pathogenesis of HF and its therapeutic potential will not be addressed here (the reader is referred to several excellent reviews [39–43] of the field). Similarly, the role of impaired calcium uptake, release, and signaling in the development of cardiac dysfunction has been comprehensively characterized [36, 37, 44]. We will focus instead on the alteration of respiratory function and its consequence, oxidative stress.

Oxidative phosphorylation represents the ultimate source of aerobic ATP production and requires the coordinated activity of the electron transport chain (ETC) consisting of enzymatic complexes I–IV and complex V (ATP synthase) at the inner mitochondrial membrane. Impairment of the ETC activity is responsible on one side for the reduced ATP generation by ATP synthase (complex V) and on the other side for the increased superoxide production mainly at complexes I and III of the ETC due to partial reduction of oxygen [45–48]. However, it has to be mentioned that mitochondria are endowed with a robust ROS-detoxifying network comprising both enzymes and nonenzymatic antioxidants that are able to counteract even a significant oxidative burden in physiological conditions. Indeed, generation of superoxide, hydroxyl anions, and hydrogen peroxide by the ETC complexes becomes relevant only in pathological conditions [49]. The term oxidative stress refers to a persistent imbalance between ROS generation and detoxification; however, the vast majority of studies have addressed the issue of ROS emission (defined as the difference between ROS production and ROS removal) without concomitant assessment of status of the antioxidant response [50].

The current evidence for ETC dysfunction and mitochondrial ROS production shows a broad variability in animal models of HF and humans with HF of different etiologies. The impairment of the ETC activity (in particular, of complexes I and III as the major sites for ROS production) in the failing myocardium has been reported in various models of HF. Ide et al. showed a decreased complex I activity with subsequent electron leakage and increased superoxide production in a model of HF induced in dogs by rapid ventricular pacing whereas the superoxide dismutase activity was not changed [51]. These authors further demonstrated in the same model a significant positive correlation between the cardiac production of superoxide and hydroxyl radicals (directly assessed by electron spin resonance spectroscopy) and the left ventricular contractile dysfunction [52]. The activities of complexes III and V have also been reported to decrease in the same experimental model of pacing-induced left ventricular failure in dogs; this paper also reported increased aldehyde levels in left failing ventricles as indirect measure of increased oxidative stress [53, 54].

In an elegant series of studies, the group of Torsten Doenst analyzed the occurrence of mitochondrial dysfunction in relation to the type of contractile abnormalities. In the rat model of HF induced by chronic pressure overload they reported a decline in complex I (but not in complex II [22]) supported respiration in isolated mitochondria that occurred in association with systolic dysfunction (diagnosed by impaired ejection fraction) 20 weeks after the induction of transverse aortic constriction (TAC) [55]. Of note, in this model, diastolic dysfunction occurred prior to the impairment of mitochondrial respiratory capacity. Interestingly, the same group recently also reported in the same experimental model (HF with systolic dysfunction 20 weeks after TAC) that the onset of diastolic dysfunction was coincident with the maximal ROS production; conversely, the occurrence of contractile dysfunction at 20 weeks was no longer related to the ROS production and was not reversed by the antioxidant interventions [56]. Similarly, in the rabbit model of pressure-overload induced HF, dysfunction of mitochondrial complexes I and II occurred during the transition from compensated left ventricular hypertrophy to overt failure and was also independent of ROS production [57]. In another experimental model of HF due to pressure overload, the spontaneous hypertensive rat, a defect in complex IV was demonstrated [58].

ETC defects were also associated with the murine model of HF induced by the coronary artery ligation. Ide et al. reported a decrease in enzymatic activity of the complexes I, III, and IV containing several mitochondrially encoded subunits (but not of the nuclear encoded complex II) and a parallel reduction in mtDNA-encoded gene transcripts, a significant increase in levels of hydroxyl radicals and lipid peroxides, changes that were associated with ventricular dilation and decreased contractility [59].

An important decrease in mitochondrial respiratory capacity was also found in a canine model of moderate HF induced by coronary microembolization in the presence of normal activities of ETC complexes, an effect that was assigned to the lack of assembly of complexes constituting the so-called respirasomes [60]. Rosca et al. considered the decrease in functional respirasomes in HF as the primary event responsible for the decreased oxidative phosphorylation and the increased ROS production leading to the progressive decline in cardiac performance [21, 61]. These authors also reported that, depending on the experimental model, mitochondrial subpopulations are differentially affected: whereas, in the canine model of intracoronary microembolization, both populations were equally affected, in the rapid ventricular pacing model, a significant decrease in oxidative phosphorylation was found in the interfibrillar mitochondria (but not in the subsarcolemmal population). Moreover, the isolation technique significantly accounts for the magnitude of the reported mitochondrial defect and explains the heterogeneity of the experimental and clinical data [21].

A great variability also characterizes the defects of ETC complexes reported to occur in the failing human heart. An important decrease of the respiratory capacity was reported in saponin-skinned muscle bundles obtained from myocardium of explanted human hearts with end-stage HF: in one study state 3 respiration was found to be significantly lower in endocardium* versus* the epicardium [62] and in the other the impairment of complex I-linked respiration was reported to occur early in the development of HF [63]. Similarly, Scheubel et al. reported a moderate decrease in complex I activity in left ventricular specimens harvested from explanted human hearts [64]; this decrease occurred in the absence of mtDNA damage, an observation that supports the hypothesis that the failing human heart is not irreversibly damaged [65]. Recently, Stride et al. reported a marked reduction in oxidative phosphorylation in left ventricle biopsies obtained from patients with chronic ischemia and systolic dysfunction (ejection fraction <45%) for complex II-supported respiration, an increased ROS production, and a tendency for decreased antioxidant defense in the ischemic tissue; however, the degree of coupling was comparable in mitochondria harvested from the ischemic and nonischemic tissue of the same heart [66]. We have previously reported that complex I- (but not complex II-) supported respiration is impaired in atrial appendages harvested from coronary patients with preserved systolic function (ejection fraction >50%) [67]. At variance from all the previous reports, in a recent study performed in freshly isolated mitochondria from failing ventricles, complex I-dependent respiration was reported to be coupled and enhanced in the failing hearts, whereas complex II-dependent succinate respiration was associated with greater uncoupling [68]. However, no major differences were found in the capacity of mitochondria to oxidize different substrates supplied* ex vivo*, a finding that reinforces the observation that reversible mitochondrial damage occurs in the failing hearts. Interestingly, these authors reported a reduced state 3 respiratory rate for succinate in the subgroup of diabetic patients, an observation suggestive for an impairment of mitochondrial respiratory capacity in the failing hearts in the presence of diabetes.

## 3. Mitochondrial Dysfunction in Diabetes

The term diabetic cardiomyopathy refers to the association of left ventricular hypertrophy/remodeling with diastolic dysfunction that precedes the development of systolic dysfunction and may progress to heart failure [69]. Elucidation of the pathogenesis of diabetic cardiomyopathy is currently an active field of research. In particular, metabolic impairment and mitochondrial dysfunction have been systematically investigated in the past decades in both clinical and experimental settings (reviewed in [70–74]). We will further refer to the impairment of respiratory capacity and the subsequent redox imbalance in order to highlight commonalities with the aforementioned findings in HF. Early studies performed in rats with type 1 DM pharmacologically induced with streptozotocin firstly mentioned the contribution of mitochondria to the diastolic dysfunction [75] and reported the decrease in succinate-supported respiration and complex II activity; the latter change was attributed to the generation of an adduct of hydroxynonenal and complex II [76]. However, most of the knowledge of mitochondrial dysfunction was gained from genetically modified rodents that recapitulate the metabolic phenotype of humans with obesity and type 2 diabetes. In spite of some differences in pathophysiological mechanisms underlying cardiomyopathy in type 1 and type 2 of experimental diabetes, compromised mitochondrial energetics is a common feature in both types of diabetes [77]. Accordingly, depressed state 3 respiration was reported to occur in experimental models of type 1 [78, 79] and type 2 diabetes [80], and also in obesity with insulin resistance [81]. In the latter study, the decrease in oxidative phosphorylation capacity was associated with increased production of H_2_O_2_ and mitochondrial uncoupling, a process that decreases cardiac efficiency and may underlie the increased propensity of diabetic hearts to develop HF [82].

As in the case of HF, whether functional differences occur in cardiac mitochondrial subpopulations has been also investigated in a murine model of type 1 diabetes [83]. Complex II-supported respiration was decreased to a greater extent in interfibrillar mitochondria (as compared to subsarcolemmal ones). In the former population, a decrease in complex I respiration was also reported together with an increased production of superoxide and a decrease in cardiolipin. However, it is not clear if the active ADP-stimulated respiratory rate as indicator of maximal respiratory capacity was also depressed in this study.

Mitochondrial dysfunction has also been confirmed in the diabetic human heart. Neufer's group reported a decreased glutamate and fatty acid-supported respiration and an increased sensitivity to Ca^2+^-induced permeability transition in permeabilized myofibers prepared from right atrial appendages harvested from coronary patients with type 2 diabetes; these authors also demonstrated the increase in oxidative stress as shown by a greater rate of H_2_O_2_ emission, glutathione depletion, and increased levels of hydroxynonenal and nitrotyrosine-modified proteins, respectively. Importantly, they also reported an inverse relationship between respiratory capacity and HbA1c [84, 85]. More recently, a decrease in complex I and fatty acid-mediated active respiration was found in subsarcolemmal (but not in interfibrillar) mitochondria isolated from atrial appendages of type 2 diabetic patients, regardless of the levels of HbA1c and hyperglycemia [86]. In another elegant study, the impairment in mitochondrial function and dynamics has been associated with contractile dysfunction in diabetic (but not in obese) patients; however, in this case, mitochondrial dysfunction correlated with the level of glycated haemoglobin [87].

The past decade of research provided convincing evidence that mitochondrial dysfunction is a central event in the pathogenesis of HF and DM. This concept extends far beyond the impairment of respiratory capacity and the generation of oxidative stress and includes several other pathomechanisms such as: impaired mitochondrial biogenesis, posttranslational modification of mitochondrial proteins, metabolic shifts and remodeling, and abnormal calcium handling that occur in both pathological conditions. Thus, it becomes more and more evident that the “common soil” hypothesis [88] proposed almost two decades ago (postulating that cardiovascular diseases and diabetes share common genetic and environmental risk factors) should be extended to include mitochondrial dysfunction as well.

## 4. Monoamine Oxidases as Novel Sources of Mitochondrial Oxidative Stress in Cardiovascular System

In the past decade, monoamine oxidases (MAOs) have emerged as another important mitochondrial source of oxidative stress in the cardiovascular system (please see [31] for a recent comprehensive review). MAOs are flavoproteins located in the outer mitochondrial membrane where they catalyze the oxidative breakdown of endogenous monoamines and dietary amines, with the constant generation of H_2_O_2_, aldehydes, and ammonia as byproducts. Two isoforms, MAO-A and MAO-B, with specific tissue distribution and substrate affinity, have been described [89]; in experimental settings, pharmacological criteria are useful to characterize the isoenzymes: MAO-A is selectively inhibited by low doses of clorgyline and MAO-B is blocked by low doses of deprenyl (selegiline) [90].

MAOs-related oxidative stress unequivocally contributes to acute myocardial ischemia/reperfusion injury [91] and to the mitochondrial dysfunction and pathologic hypertrophy elicited by pressure overload in a murine model of HF [92, 93]. Of note, MAO-A protein has been reported to be overexpressed in all the experimental models of HF induced in rat by hemodynamic overload (pressure and volume) and coronary ligation [31]. Also, MAO-A activity has been reported to increase in response to angiotensin II, an observation relevant for the clinical settings of heart failure and diabetes where the renin-angiotensin system is upregulated [94].

Also, MAOs have emerged as mediators of experimental endothelial dysfunction via the excessive H_2_O_2_ production in two murine models of acute (induced with lipopolysaccharide, LPS) and chronic (induced with angiotensin II and A II) oxidative stress, respectively [12]. In this study, we demonstrated that exposure of mouse aortas isolated in organ bath to* exogenous* MAO elicited endothelial function via a ROS-dependent mechanism. Importantly, both the impairment of endothelial-dependent relaxation and H_2_O_2_ emission were partially reversible in the presence of pharmacological inhibition of MAO-A (with clorgyline and moclobemide) and MAO-B (with selegiline), respectively. Importantly, in this model, endogenous vascular catecholamines are sufficient to activate MAO to induce endothelial dysfunction (no exogenous substrate was added in the experiment). The mechanism was most probably related to the decreased vascular generation of nitric oxide since in a separate set of experiments MAO-A was found to limit the endothelial accumulation of cyclic guanosine monophosphate. We further investigated, in organ bath experiments, the contribution of* endogenous* MAO as mediator of endothelial dysfunction. We found that both MAO isoforms are expressed in the vascular system and induced in response to LPS and A II via the NF-*κ*B and phosphatidylinositide 3-kinase signaling [95].* In vivo* exposure to A II and LPS increased MAO expression in aortic rings and acute MAO inhibition partially restored normal endothelium-dependent relaxation in vessels harvested from A II and LPS treated animals; this effect was associated with a reduction in the vascular formation of H_2_O_2_ [12].

**Figure 1:**
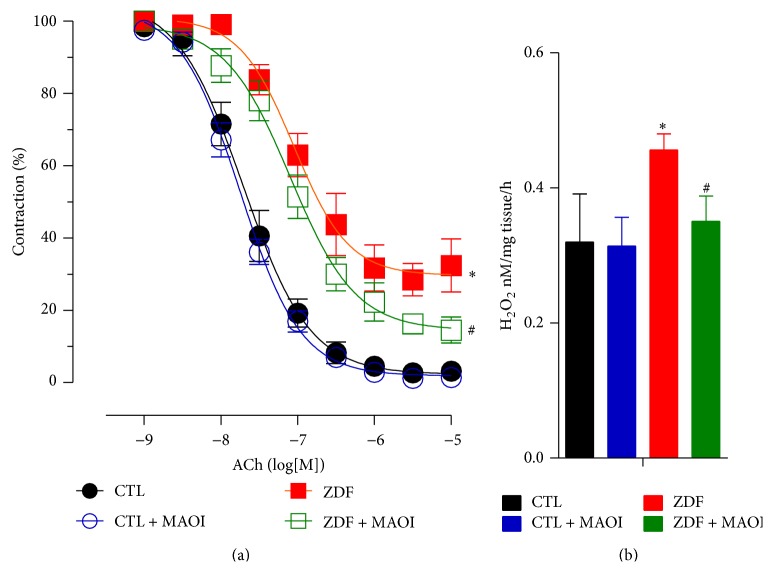
Effects of MAO-A inhibition on vascular function in isolated rat aortas. (a) Acetylcholine-induced endothelium-dependent relaxation in phenylephrine-preconstricted aortic segments (*n* = 4, ^*∗*^ *P* < 0.05 with and without diabetes; ^#^ *P* < 0.05 with and without MAO inhibitor, clorgyline, 10 *μ*mol/L). (b) Assessment of H_2_O_2_ formation by ferrous oxidation xylenol orange (FOX) assay in the presence or absence of the MAO inhibitor (*n* = 4, ^*∗*^ *P* < 0.05 with and without diabetes; ^#^ *P* < 0.05 with and without clorgyline, 10 *μ*mol/L).

We also recently demonstrated that MAO-A inhibition corrects endothelial dysfunction in Zucker diabetic fatty rat (ZDF), a genetic model of type 2 diabetes [96]. In organ bath experiments, preincubation for 30 min with the MAO-A inhibitor, clorgyline, significantly improved the endothelium-dependent relaxation of the aortic rings isolated from ZDF rats and had no effect on vascular relaxation in control aortic rings. Also, vascular H_2_O_2_ generation was increased in diabetic vessels and significantly decreased in the presence of clorgyline (10 *μ*mol/L, Figure 1).

Whether basic science's predictions on the role of MAO inhibition in the failing heart hold true in humans is not known. A pioneering study has recently reported that atrial activity of MAO assessed in right atrial appendages may serve as an independent predictor for postoperative atrial fibrillation in patients undergoing cardiac surgery [97].

Eugene Braunwald pointed out already back to 1997 that there are two emerging epidemics of cardiovascular disease, heart failure and atrial fibrillation [98]. MAOs contribution to both conditions has been documented. In line with our experimental data, it is conceivable to address the role of the enzyme in DM which together with obesity are the other two menacing pandemics of the 21st century. Accordingly, contribution of MAO-related oxidative stress to the pathogenesis of endothelial, mitochondrial, and contractile dysfunction in diabetic patients undergoing cardiac surgery should be thoroughly investigated.

Moreover, several studies reported the contribution of NADPH oxidase and eNOS uncoupling to the pathological production of vascular ROS after percutaneous coronary interventions (reviewed in [99]). In line with previously reported contribution of MAOs to the experimental endothelial dysfunction it is tempting to speculate that MAO-derived ROS may be involved in the postprocedural complications such as restenosis and stent thrombosis.

## 5. Conclusions

The past decade of research provided convincing evidence that mitochondrial dysfunction may be an important event in the development of pathological hypertrophy in both heart failure and diabetic cardiomyopathy. Not only mitochondrial but also endothelial dysfunction is a widely investigated mechanism in cardiometabolic diseases and a valuable therapeutic target. There is an unmet need for novel therapies tailored to reduce the risk of heart failure in patients with diabetes mellitus. Therefore, the design of a prospective study in cardiac patients with and without diabetes undergoing heart surgery aimed at providing further mechanistic insights into the role of MAO as an emerging mitochondrial therapeutic target for cardio- and vasculoprotection is strongly recommended.
